# Oral pimonidazole unveils clinicopathologic and epigenetic features of hypoxic tumour aggressiveness in localized prostate cancer

**DOI:** 10.1186/s12885-024-12505-1

**Published:** 2024-06-18

**Authors:** Xinpei Ci, Sujun Chen, Rui Zhu, Mojgan Zarif, Rahi Jain, Wangyuan Guo, Matthew Ramotar, Linsey Gong, Wenjie Xu, Olivia Singh, Sheila Mansouri, Gelareh Zadeh, Gong-Hong Wei, Wei Xu, Robert Bristow, Alejandro Berlin, Marianne Koritzinsky, Theodorus van der Kwast, Housheng Hansen He

**Affiliations:** 1 Princess Margaret Cancer Centre, University Health Network, Toronto, ON Canada; 2 Present Address: West China School of Public Health, West China Fourth Hospital, and State Key Laboratory of Biotherapy, Sichuan University, Chengdu, Sichuan China; 3 Department of Medical Biophysics, University of Toronto, Toronto, ON Canada; 4 MOE Key Laboratory of Metabolism and Molecular Medicine and Department of Biochemistry and Molecular Biology of School of Basic Medical Sciences, and Fudan University Shanghai Cancer Center, Shanghai Medical College of Fudan University, Shanghai, China; 5 Division of Neurosurgery, Department of Surgery, University of Toronto, Toronto, ON Canada; 6 Division of Cancer Sciences, University of Manchester, Manchester, UK; 7 Christie NHS Trust and CRUK Manchester Institute and Cancer Centre, Manchester, UK; 8 Department of Radiation Oncology, University of Toronto, Toronto, ON Canada; 9 Institute of Medical Science, University of Toronto, Toronto, ON Canada; 10 Division of Anatomic Pathology, Laboratory Medicine Program, University Health Network, Toronto, ON Canada

**Keywords:** Prostate Cancer, Hypoxia, DNA Methylation, PIMO, Biomarker

## Abstract

**Background:**

Tumor hypoxia is associated with prostate cancer (PCa) treatment resistance and poor prognosis. Pimonidazole (PIMO) is an investigational hypoxia probe used in clinical trials. A better understanding of the clinical significance and molecular alterations underpinning PIMO-labeled tumor hypoxia is needed for future clinical application. Here, we investigated the clinical significance and molecular alterations underpinning PIMO-labeled tumor hypoxia in patients with localized PCa, in order to apply PIMO as a prognostic tool and to identify potential biomarkers for future clinical translation.

**Methods:**

A total of 39 patients with localized PCa were recruited and administered oral PIMO before undergoing radical prostatectomy (RadP). Immunohistochemical staining for PIMO was performed on 37 prostatectomy specimens with staining patterns evaluated and clinical association analyzed. Whole genome bisulfite sequencing was performed using laser-capture of microdissected specimen sections comparing PIMO positive and negative tumor areas. A hypoxia related methylation molecular signature was generated by integrating the differentially methylated regions with previously established RNA-seq datasets.

**Results:**

Three PIMO staining patterns were distinguished: diffuse, focal, and comedo-like. The comedo-like staining pattern was more commonly associated with adverse pathology. PIMO-defined hypoxia intensity was positively correlated with advanced pathologic stage, tumor invasion, and cribriform and intraductal carcinoma morphology. The generated DNA methylation signature was found to be a robust hypoxia biomarker, which could risk-stratify PCa patients across multiple clinical datasets, as well as be applicable in other cancer types.

**Conclusions:**

Oral PIMO unveiled clinicopathologic features of disease aggressiveness in localized PCa. The generated DNA methylation signature is a novel and robust hypoxia biomarker that has the potential for future clinical translation.

**Supplementary Information:**

The online version contains supplementary material available at 10.1186/s12885-024-12505-1.

## Introduction

Prostate cancer (PCa) is the second most common cancer worldwide in men [1]. Patients with indolent disease can live for years without progression, while aggressive disease can quickly metastasize thus becoming incurable. Clinical stage (T-category), biopsy Gleason score and serum prostate-specific antigen (PSA) level are used to stratify localized PCas into low-, intermediate- and high-risk groups [2–4]. However, significant clinical heterogeneity remains within these clinicopathologic-defined risk groups, reflected in variable rates of disease recurrence following curative-intent treatment [5]. This heterogeneity has been related to additional genomic [6] and/or clinicopathologic features of aggressiveness at diagnosis, such as intraductal carcinoma or cribriform architecture (IDC/CA) [7], and to the existence of subpopulations of radioresistant tumor cells or micro-metastatic disease at the time of primary treatment [8]. Improving individualization of therapies and outcomes hinges on the discovery of novel clinical biomarkers to better identify and risk-stratify patients with aggressive disease.

Hypoxia is a critical feature of the heterogeneous PCa tumor microenvironment [9, 10]. It has a central role in tumor progression and treatment response, and serves as an independent predictor of biochemical recurrence after radical radiotherapy and prostatectomy [5, 11, 12]. The exogenous hypoxia marker pimonidazole (PIMO) is a 2-nitroimidazole compound that is selectively reduced and covalently bound to intracellular macromolecules in areas of hypoxia (pO2 < 10 mm Hg) within normal and tumour tissue with current approval for investigational clinical use [9, 13, 14]. Gene expression signatures derived from bulk RNA-seq of PIMO stained radical prostatectomy (RadP) specimens have emerged as biomarkers indicating disease prognosis [9, 15]. Considering the intratumoral heterogeneity and the diversity of gene expression regulation, we expanded the biomarker analysis to the epigenetic level, as manifested by DNA methylation, and improved the resolution to tissue cellular spatial level, as achieved by laser capture microdissection (LCM).

In the present clinical study (from 24/03/2014 to 20/12/2018), thirty-nine patients with intermediate/high-risk PCa undergoing an open RadP were administered PIMO prior to surgery. Carcinoma and benign prostatic hyperplasia (BPH) samples were collected from the surgical specimens. We used the BPH samples as tumor negative controls as benign tissues are likely to be contaminated by carcinomas. More importantly, BPH is also associated with hypoxia but is not pre-malignant like high-grade prostatic intraepithelial neoplasia [16]. Immunohistochemical staining for PIMO was performed on most representative formalin fixed paraffin sections and assessed microscopically for staining pattern and intensity. PIMO positive tumor areas (i.e. hypoxic region), PIMO negative tumor areas (i.e. normoxic region), and BPH areas were isolated using LCM for whole-genome bisulfite sequencing (WGBS) to analyze the DNA methylation status. A hypoxia related methylation molecular signature was generated by integrating the differentially methylated regions with our previously established RNA-seq datasets [17]. This signature robustly risk-stratified PCa patients across several distinct publicly available clinical datasets, and was also applicable in other cancer types.

## Results

### Oral pimonidazole in intermediate/high risk prostate cancer patients undergoing radical prostatectomy

**Fig. 1:**
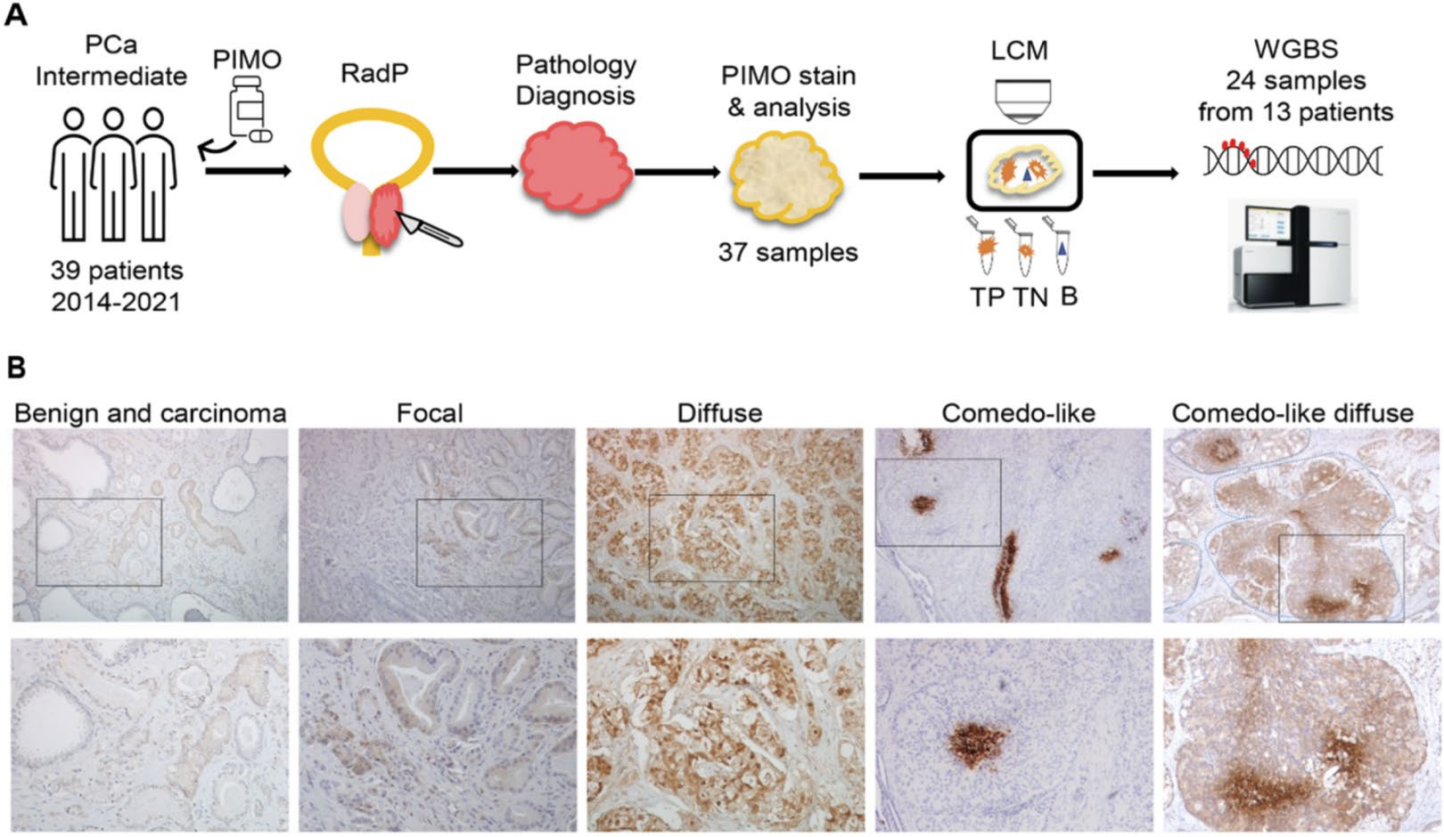
PIMO-labelled hypoxia in patients undergoing radical prostatectomy. **A** Graphical schematic of the study design. 39 patients were accrued to the trial, with 37 specimens processed with PIMO staining, and 24 samples from 13 patients underwent LCM and WGBS. RadP, radical prostatectomy. LCM, laser capture microdissection. TP, tumor PIMO positive; TN, tumor PIMO negative; B, Benign. WGBS, whole genome bisulfite sequencing. **B** Microscopic images showing immunohistochemical PIMO labeling patterns of prostate cancer. From left to right: Diffuse PIMO labeled carcinoma glands intermingled with PIMO negative benign glands; Few PIMO-positive carcinoma glands surrounded by larger area of PIMO-negative adenocarcinoma; Confluent area of carcinoma, intensely labeled by PIMO; Intense PIMO central plug of vital carcinoma surrounded by unlabeled sheet of carcinoma with features of cribriform carcinoma; Intraductal carcinoma, diffusely labeled by PIMO with central more intensely labeled plugs. Cribriform structure is circled with blue dashed lines. The lower panel shows the magnifications of the selected regions picked from the upper panel

To evaluate intratumoral hypoxia in clinical prostate carcinoma by PIMO labeling, carcinoma and BPH samples were collected from patients who consented for this Institutional REB approved clinical trial (13–7172-C, https://clinicaltrials.gov/ct2/show/NCT02095249) and underwent RadP, as part of standard of care treatment at a Comprehensive Cancer Centre. Thirty-nine patients, from 24/03/2014 to 20/12/2018, were accrued with the following eligibility criteria: intermediate- or high-risk group, clinical stage T2-T3 N0 M0, pathology of adenocarcinoma of the prostate, and Gleason score 7 (biopsy ISUP GG 2–3) with > 40% biopsies involved with tumour or Gleason score 8–10 (biopsy ISUP GG 4–5) regardless of the percentage of biopsies involved. All patients received a single oral dose of pimonidazole (0.5 g/m^2^) 24 h prior to surgery. Within 30 min of prostatectomy, fresh tissue samples of visible carcinoma and BPH (~ 8mm^3^) were isolated from the extracted prostate by the study pathologist (Fig. 1A). The remainder of the prostatectomy specimens were fixed in formalin, grossed and processed according to standard procedures of the pathology department. Haematoxylin–eosin stained paraffin sections were examined for carcinoma and most representative sections in terms of tumour extent and Gleason score were selected for PIMO staining. Paraffin sections were stained for PIMO with pattern and intensity evaluated and scored by the study uropathologist (TvdK; Fig. 1A). Baseline characteristics of all 39 patients in the trial are summarized in Tables 1 and S1. Median follow-up after RadP was 44 months (range 1–80). No PIMO-attributable adverse events were observed.

**Table 1:** Baseline characteristics of all patients in the trial

Variable	All patients (*n* = 39)
Age, years (median (range))	70.4 (56.3–86.4)
Pre-RP PSA, ng/ml (median (range))	7.4 (2.0–25.4)
Prostatic intraepithelial neoplasia (%)	19 (48.7%)
Positive surgical margins (%)	14 (35.9%)
pN1 (%)	4 (10.3%)
Lymphovascular invasion	5 (12.8%)
IDC/CA presence (%)	17 (43.6%)
Follow-up, months (median (range))	44 (1–80)
RP ISUP GG number (%)	
1	2 (5.1%)
2	12 (30.8%)
3	15 (38.5%)
4–5	10 (25.6%)
Events number (%)	
BCR	8 (20.5%)
Metastasis	1 (2.6%)

*RP* radical prostatectomy, *ISUP GG* International Society of Urological Pathology grade group, *pN1* lymph node invasion, *BCR* biochemical relapse, *IDC/CA* intraductal carcinoma and/or cribriform architecture, *ng/ml* nanograms per milliliter

### PIMO staining detected distinct hypoxia patterns and association with clinical outcomes

PIMO staining was evaluated in 37 hormone-naive cases (one case was excluded due to missing blocks and one due to use of neoadjuvant androgen deprivation therapy and extensive inflammatory changes). Absent or very faint staining was considered as negative. In 26 of the 37 cases (70%) PIMO staining was noted. Taking into account the underlying carcinoma architecture, three staining patterns could be distinguished, that is: diffuse, focal, and comedo-like (Fig. 1B). Diffuse staining was generally of moderate intensity decorating patchy or larger confluent areas, involving > 5% of carcinoma. Focal staining was moderate to intense, labeling less than 5% of the conventional acinar adenocarcinoma area. Comedo-like staining was a focal often intense staining seen centrally in larger sheets and nodules of carcinoma without intervening stroma, resembling the necrotic plugs as in comedocarcinoma. If a histology of comedonecrosis was present, carcinoma cells surrounding the necrotic plug were PIMO-positive. Comedo-like staining could be isolated but was mostly in conjunction with diffuse staining (Figs. 1B, S1).

The distribution of PIMO staining patterns is listed in Table S2. The majority (62%) of PIMO-positive cases showed an exclusive diffuse staining pattern and a comedo-like pattern was seen in nine cases (35%). The latter pattern was only identified in cases with IDC/CA (Figs. 1B, S1), two histopathological features frequently associated with high-grade cancer and poor prognosis [18, 19]. Among the 14 low grade (GG 1–2) carcinomas, four were PIMO-negative (29%), one showed a comedo-like pattern (7%), one a focal pattern (7%) and eight a diffuse pattern (57%). Among the 23 high grade (GG 3–5) carcinomas, seven were PIMO-negative (30%), eight a comedo-like pattern (35%), one a focal pattern (4%) and seven showed a diffuse (non-comedo-like) pattern (30%). Five of the eight high grade carcinomas displaying comedo-like patterns were GG4 or GG5 carcinomas. Among the 17 IDC/CA positive cases the majority showed a comedo-like pattern (53%), and in only 3 out of the 17 cases (18%) no PIMO staining was observed (Table S3).

We next analyzed how the PIMO staining patterns and intensities correlated with clinico-pathological features. As summarized in Tables 2 and S4, PIMO intensity was significantly associated with pathologic T (pT)-category (*p =* 0.047), lymphovascular invasion (LVI) (*p =* 0.011), Gleason score (*p =* 0.003), number of positive margins (*p =* 0.031), extraprostatic extension (*p =* 0.038), and presence of IDC/CA morphology (*p =* 0.002). PIMO staining pattern was also significantly associated with adverse pathology (Table 2), in particular, the comedo-like PIMO pattern correlated with pathologic T (pT)-category (*p =* 0.024) and LVI (*p =* 0.006) (Table S5). Our findings suggest that hypoxia unveiled by PIMO staining is correlated with features of tumor aggressiveness, in line with previous studies [9, 15].

**Table 2:** Correlation of PIMO pattern and intensity with clinical features

Clinical features	PIMO pattern		PIMO intensity	
	Association	*p* value	Association	*p* value
Pathologic T-category	0.56	**0.005**	0.11	**0.047**
LVI	0.49	**0.001**	0.41	**0.011**
Sum Gleason score	0.27	**0.019**	0.48	**0.003**
Number of positive margins	0.41	0.071	0.58	**0.031**
Extraprostatic extension	0.31	**0.011**	0.34	**0.038**
IDC/CA	0.63	**0.001**	0.2	**0.002**

*PIMO* pimonidazole, *LVI* lymphovascular invasion, *RP* radical prostatectomy, *IDC/CA* intraductal carcinoma and/or cribriform architecture

### Whole genome bisulfite sequencing of PIMO+ and PIMO- specimens

**Fig. 2:**
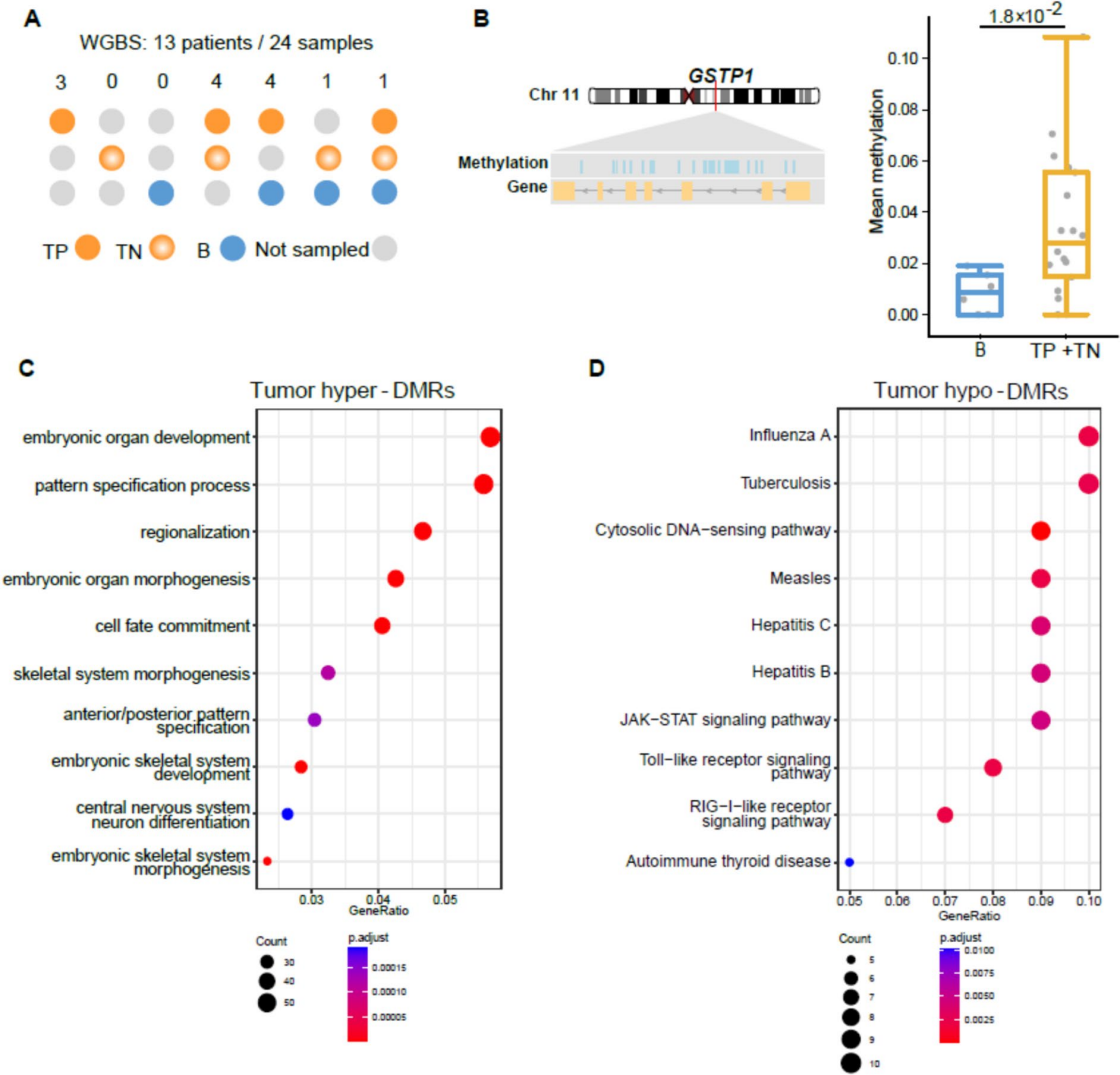
Whole genome bisulfite sequencing. **A** Sampling schematics for the WGBS cohorts. The number above shows the number of patients subjected to the indicated sampling strategy. **B** Example of a hypermethylated gene, *GSTP1*, identified in tumor samples (TP + TN). Left, genome track and hypermethylated loci of *GSTP1*. Right, box plot of mean methylation levels for the indicated samples. P value was calculated by Mann–Whitney test. **C-D** Pathways and terms enriched by genes harboring hyper-DMR (**C**) and hypo-DMR (**D**) in comparision of the tumor vs. benign samples

To characterize the methylation profile changes in hypoxic tumors of PCa, we curated a total of 24 paraffin-embedded samples from 13 PCa patients (three and ten RP ISUP GG 1–2 and 3–5, respectively, Table S1) using laser capture microdissected tissues, including 12 hypoxic tumors with a representative distribution of PIMO staining patterns, 6 normoxic tumors and 6 normoxic benign samples (Fig. 2A, Table S1). DNA extracted from the 24 samples were subjected to bisulfite conversion and deep sequencing, with a median of 350 M total reads per sample, of which 79% on average are uniquely mapped and 72% methylated cytosine in CpG are captured (Table S6). Principal component analysis revealed notable inter-patient variations, with PC1 associated with Gleason score, and PC2 distinguishing benign and tumor samples (Figure S2A). We next pooled the 12 PIMO positive and 6 negative tumor samples to compare with the 6 benign samples and defined tumor-specific differentially methylated regions (DMRs), including 6,150 hypo- and 4,775 hyper-DMRs. These tumor specific hyper-DMRs are enriched in CpG island (CGI) and shores while in sharp contrast, the hypo-DMRs are enriched in open sea regions (Figure S2B-C). A previously reported hypermethylated gene in PCa, *GSTP1*, was also captured (Fig. 2B). GO analysis of the genes harbouring hyper-DMRs are enriched in developmental and cell fate commitment related terms, consistent with cancer cells being generally poorly differentiated (Fig. 2C). Interestingly, enriched terms and pathways associated with hypo-DMRs are immune-related, including the Toll- and RIG-I- like receptor signaling, and the cytosolic DNA-sensing pathway that can activate the cGAS-STING signaling (Fig. 2D). Comparison of the DMRs between carcinomas and benign tissues identified in this study with previously reported DMRs from an Asian cohort of WGBS revealed that 27.5% (*P* value is 1.0 × 10^–4^) of hyper-DMRs and 12.1% (not significant) of hypo-DMRs were overlapping (Figures S2D-E), consistent with the observation in the Asian cohort that hypermethylation tends to be more recurrent than hypomethylation [20]. These results corroborate the validity of our analysis.

### Identification of DNA methylation marker for tumor hypoxia

**Fig. 3:**
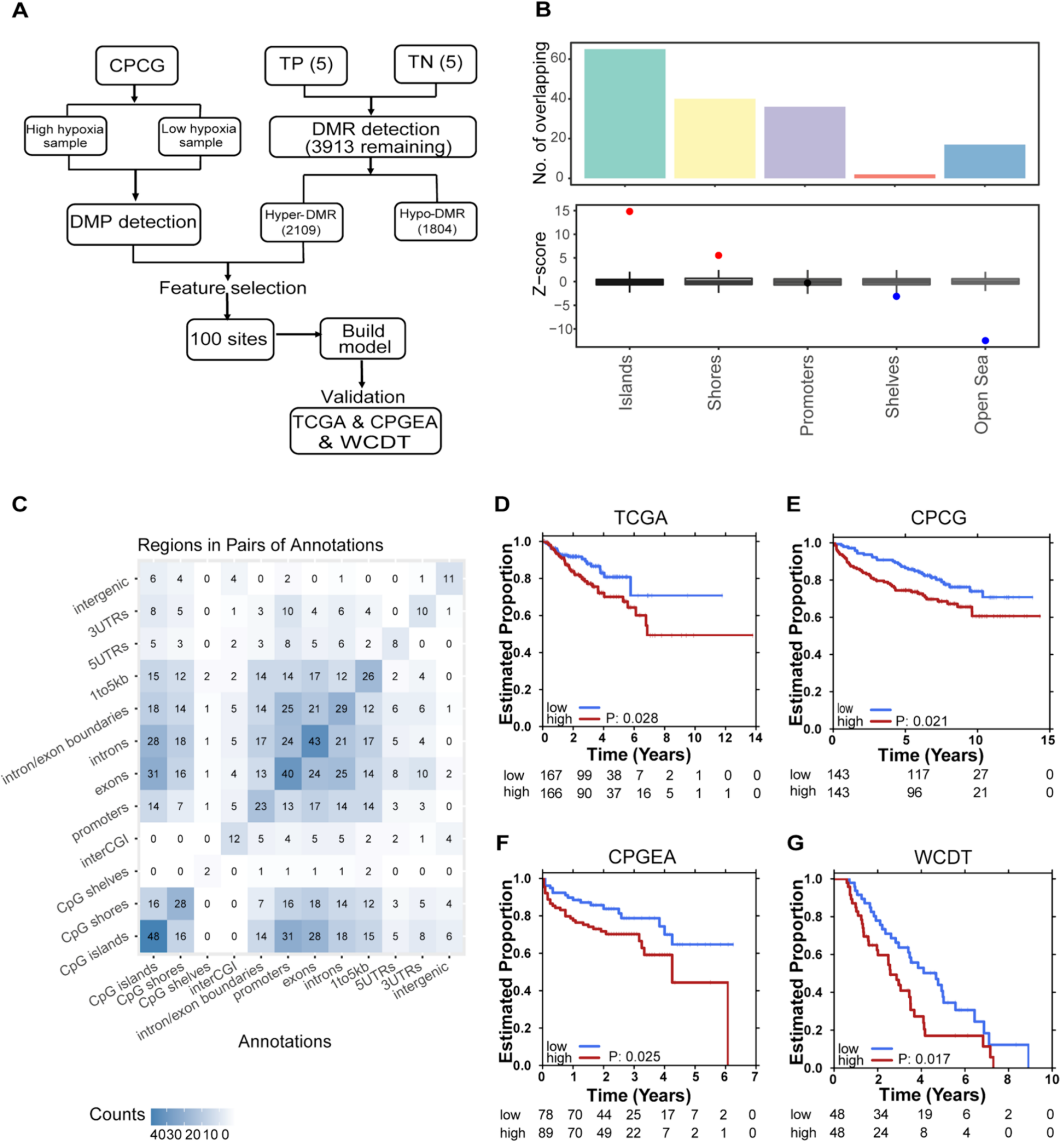
Integrated hypoxia biomarker. **A** Analysis pipeline of methylation hypoxia scores. **B** Annotation of the 100 hypoxia signature hyper-DMRs on genomic regions. **C** Correlation of the 100 hypoxia signature hyper-DMRs distributed on genomic regions. **D-G** Survival rates for the high and low groups of patients, stratified using the hypoxia DMR signature score in various clinical cohorts. Progression-free (PFS) survival was used in the TCGA cohort (**D**); biochemical recurrence-free (BCR) survival was used in the CPCG (**E**) and CPGEA (**F**) cohorts; and overall survival (OS) was used in the WCDT cohort (**G**)

We next sought to identify hypoxia associated DMRs by comparing Tumor-PIMO Positive (TP) regions (i.e. hypoxic tumor) with Tumor-PIMO Negative (TN) regions (i.e. normoxic tumor). Five patients had paired hypoxic tumor and normoxic tumor samples profiled, based on which we defined hypoxia associated DMRs, including 1,804 hypo-DMRs, and 2,109 hyper-DMRs (Fig. 3A). While no obvious enrichment was observed for the hyper-DMRs, the hypo-DMRs are enriched in open sea regions (Figure S3A-B). No enriched GO terms were identified for genes nearby these DMRs, likely due to the lack of enrichment at promoter regions and small number of DMRs detected.

Hypoxia is associated with worse outcome in multiple cancer types [10]. Several RNA hypoxia signatures are reported to be able to distinguish disease outcomes [9, 21]. However, RNA expression is transiently regulated and may be more susceptible to temporal variability that affects their performance as biomarkers (Table S7). Epigenetic marks, in particular, DNA methylation are more stably maintained and inherited, and thus potentially may represent a more effective choice for clinical application. While DNA methylation hypoxia markers from cell models have been reported [22], in vitro environments cannot fully recapitulate patient scenarios. DNA methylation hypoxia markers derived directly from patient samples have not yet been defined. However, similar as the RNA hypoxia score, the hypoxia hyper-DMRs and hypo-DMRs were associated with clinical outcome in only one out of the four cohorts tested (Table S7).

To further curate a robust DNA methylation hypoxia marker, we integrated the RNA hypoxia marker with the hypoxia DMRs in the Canadian Prostate Cancer Genome (CPCG) network cohort [17]. Briefly, samples were first grouped into high and low groups according to the Ragnum RNA hypoxia score [9] calculated using the RNA-seq data of the CPCG cohort [17]. Then, differentially methylated probes between the Ragnum high and low groups were identified using the methylation array data of the CPCG cohort. Finally, the hypoxia-associated hyper-DMRs from the WGBS data of this study overlapping with probes with higher methylation in the high Ragnum score group from the CPCG datasets were retained (Fig. 3A). This analysis identified a total of 100 overlapping hyper-DMRs, which are enriched in CpG islands and shores while depleted in open sea regions (Fig. 3B-C). The average methylation levels of the 100 overlapping hyper-DMRs were used to develop a novel methylation hypoxia signature score. This methylation hypoxia signature is associated with clinical outcome in all the four cohorts tested, with higher methylation hypoxia signature score associated with shorter progression-free survival in the Cancer Genome Atlas (TCGA) cohort [23], increased disease recurrence rates in the CPCG and the Chinese Prostate Cancer Genome and Epigenome Atlas (CPGEA) [20] cohorts, and shorter overall survival in the West Coast Prostate Cancer Dream Team (WCDT) [24] cohort (Figs. 3D-G, S4). We then expanded the analysis to include other cancer types in the TCGA cohort. Significant overall survival associations in the Low Grade Glioma (LGG, *p =* 3.88 × 10–5) [25], the Kidney Renal Clear Cell Carcinoma (KIRC, *p =* 0.0044) [26] and the Glioblastoma (*p =* 0.054) datasets [27] (Table S8) were observed, suggesting the potential of capturing common hypoxia features using the methylation signature.

## Discussion

Hypoxia is frequently observed in PCa, and is associated with disease recurrence after local treatment, treatment resistance and metastatic disease. This clinical study demonstrates two major findings: 1) the oral hypoxia probe, PIMO, can be used to identify staining patterns and intensities that have relevance with other histopathological features and prognostic factors; 2) the novel DNA methylation signature of hypoxia can robustly risk-stratify PCa patients.

By utilizing whole sections of prostatectomy specimens, a few distinct PIMO-labelling patterns were observed, including diffuse, focal and comedo-like. The different PIMO-labeling patterns may suggest clinical significance. The comedo-like hypoxia pattern is first described in this study. The term comedo-like refers to its similarity with histopathological features of comedocarcinoma, the most aggressive Gleason pattern 5 sub-pattern [28], mainly seen in IDC [29]. Consistently, the comedo-like pattern is exclusively observed in IDC/CA samples in our series, and this pattern is significantly correlated with worse disease stages and lymphovascular invasion. This observation is in line with a recent study that IDC with comedonecrosis conveys a worse prognosis as compared to IDC without comedonecrosis [30]. As such, comedo-like PIMO labeling may precede or corroborate the H&E-depicted comedonecrosis defining comedocarcinoma of the prostate. Previous studies have reported that PIMO staining positivity percentage and intensity are positively correlated with tumor aggressiveness, in particular, tumor stage and lymph node metastasis [9, 15]. This is also the case in our cohort. An interesting finding is that PIMO intensity is significantly correlated with IDC/CA morphology. We also observed that both the PIMO staining pattern and intensity are significantly correlated with the pathologic T-category and IDC/CA. However, the association with the pattern is much stronger than with the intensity. This may be due to the exclusive appearance of the comedo-like pattern in more advanced disease and IDC/CA cases. Therefore, for future clinical applications of PIMO staining, identifying the PIMO staining pattern in addition to its intensity could potentially provide additional diagnostic and prognostic information. IDC/CA is a unique histological pattern significantly associated with lymph node metastasis and poor prognosis [19, 31, 32]. Our early study also found that carcinomas with IDC/CA patterns had higher hypoxia corresponding with increased disease recurrence and resistance to therapy [33]. An explanation for the higher hypoxia status in cribriform pattern in IDC/CA is its three-dimensional structure devoid of intervening stroma or vasculature. Therefore, tumour cells at the centre of a cribriform architecture are exposed to restricted amounts of oxygen that diffuses through to the inner cell mass of this dense glandular architecture. The architecture of most other prostate morphologies comprises tumor cells in direct contact with stroma, thereby, limiting this hypoxia eliciting phenomenon [34, 35]. This plausible causality and its clinical significance between IDC/CA morphology and hypoxia deserves further studies. On the other hand, a non-negligible proportion of IDC/CA pattern carcinoma lacked PIMO staining. It would be interesting to compare the PIMO positive to negative IDC/CA tumors or regions to uncover the biological heterogeneity among these cancers which could imply a different propensity to metastatic disease. Alternatively, limited drug distribution (e.g., PIMO hardly penetrating a very hypoxic/hypovascular region) might be another potential reason which is worth further investigation.

Hypoxia is associated with tumor prognosis [36, 37], nevertheless, previous gene expression based signatures showed variable performance in different clinical cohorts [10]. Hypoxia can directly affect DNA methylation [38], and epigenetic modifications usually precede alterations in gene expression. In this work, we applied LCM technique on tumor areas to analyze the genome wide methylation change between hypoxic and normoxic regions. This was the first WGBS study of PIMO-defined hypoxia in PCa. We unveiled a hypoxia-related DNA methylation signature consistently associated with disease outcomes across multiple PCa datasets. Notably, while the signature was generated using Canadian-based patient collections, it was validated with patients from different origins, including Asian patients (CPGEA). This result suggests that hypoxia-related methylation can robustly reflect tumor microenvironment alterations, regardless of the patient's germline background. Hypoxic tumor microenvironment associated DNA methylation alterations have been previously observed in various other cancer types, such as breast cancer [39], hepatocellular carcinoma [40] and lung cancer [41]. Our DNA methylation signature was also able to discern disease prognosis in other cancer types including glioma and kidney cancer. From a mechanistic point of view, the hypoxic microenvironment is fundamentally linked with aberrant DNA methylome. The activity of ten-eleven translocation (TET) methylcytosine dioxygenases that initiates the demethylation of DNA is directly dependent on oxygen level [42]. Hypoxia-induced loss of TET activity thus increases hypermethylation [43, 44]. This hypoxia/TET/hypermethylation axis may explain why a hypermethylation signature rather than a hypomethylation signature can consistently stratify patients’ risk in multiple cohorts and cancer types. It is still instrumental to investigate how precisely hypoxic environments in prostate tumors and particularly in special histopathological structures such as IDC/CA affect specific gene methylation. Considering the higher stability of DNA methylation compared to RNA features, our hypoxia methylation signature may also be potentially applied in liquid biopsy which has made tremendous progress in translational medicine in the last decade.

## Methods

### Human specimens

All patients (*n* = 39) were recruited under clinical study https://clinicaltrials.gov/ct2/show/NCT02095249, provided informed consent and all samples were obtained upon approval of the institutional ethics committee and Research Ethics Board at the University Health Network (UHN). CONSORT guidelines were used [45]. All PCa patients had already agreed to undergo an open radical prostatectomy at Princess Margaret Cancer Centre-UHN with bulky intermediate risk or high-risk disease (clinical stage T2-T3 N0 M0, pathology of adenocarcinoma of the prostate, Gleason score 7 with > 40% biopsies involved with tumour or Gleason score 8–10 with any percentage of involved biopsies).

Pimonidazole has been approved for use in PCa patients at Princess Margaret Cancer Centre by Health Canada (Control No 159538, UHN REB# 12–5015-C). Patients were instructed to take the pimonidazole orally at 0.5 g/m^2^ between 12–1 pm the day prior to the surgery (e.g., approximately 24 h before surgery). Both tumor and BPH samples were collected from patients. Haematoxylin and eosin (HE)-stained whole-mount sections of 5 μm thickness were used for histopathological staging and grading by a pathologist (TvdK.).

### Laser capture microdissection

Sections of 10-micron thickness were cut on membrane slides which were lightly counterstained with haematoxylin for visualization of the different areas. As a template to distinguish PIMO-positive and negative tumour areas, we used PIMO- and H&E stained corresponding slides. Laser capture microdissection was performed using the Leica LMD7000 equipment. Microdissected samples were collected in Eppendorf vials, buffer was added and stored at 4 °C until the samples were submitted for DNA extraction and analysis by Oncoscan.

### Immunohistochemistry

Formalin-Fixed Paraffin-Embedded (FFPE) sections were deparaffinized in xylene and hydrated in progressively diluted ethanol solutions. Heat-induced antigen retrieval (HIER) was performed using a sodium citrate dehydrate buffer pH 6. Endogenous peroxidase activity was inhibited by incubating sections in 3% H_2_O_2_ in methanol. Slides were incubated for one hour at room temperature in a blocking solution of 5% BSA in 1X PBS with 0.1% Triton X-100. Slides were incubated in primary antibody against PIMO (Hypoxyprobe 4.3.11.3) in a 1:1000 dilution of blocking buffer overnight at 4 °C, followed by incubation with secondary antibody (EnVision + System HRP Labelled Polymer anti-mouse K4000) for one hour at room temperature. Immunohistochemical staining was performed using the Vector® DAB Peroxidase Substrate kit (SK-4100, Vector Laboratories). Sections were counterstained with hematoxylin, dehydrated in progressively stronger ethanol solutions, and cover slipped. The pattern and intensity of PIMO staining were evaluated blindly by a pathologist (TvdK.).

### DNA isolation

DNA extractions were done using AllPrep DNA/RNA FFPE Kit (Qiagen, CA) according to manufacturer’s protocol. Briefly, samples were deparaffinized in xylene at 50 °C, deparaffinized tissue pellets were incubated at 56 °C for 15 min in a proteinase K containing buffer. The supernatant was incubated in a proteinase K containing buffer for 1 h at 56 °C, then 2 h at 90 °C. Samples were eluted using QIAmp MinElute spin column according to protocol.

### Publicly available data

The TCGA prostate adenocarcinoma (PRAD) 450 K methylation data (hg19 based) were downloaded from the TCGA Data Portal (https://tcga-data.nci.nih.gov/tcga/), including 50 normal tissue and 489 primary tumor samples. Associated clinical data and normalized gene expression were also obtained. The CPCG (Canadian Prostate Cancer Genome) 450 K methylation data from 286 patients with localized prostate adenocarcinoma, matching normalized gene expression and clinical information (hg19 based) were obtained from previous publication [46]. Processed whole-genome bisulfite sequencing (WGBS, hg38-based), RNA-seq and clinical data for 194 Asian patients with localized tumors and matched healthy tissue were obtained from CPGEA, the Chinese Prostate Cancer Genome and Epigenome Atlas (http://www.cpgea.com) [20, 47]. Processed WGBS and matched RNA-seq data (hg38-based) for 100 WCDT mCRPC (West Coast Dream Team, metastatic castration-resistant PCa) were obtained from previous publication [24]. The hg38 genome coordinates were converted to hg19 using liftOver (v1.18.0) R package with a chain file retrieved from the UCSC genome browser (https://genome.ucsc.edu/).

### Sequencing data preprocessing

FastQC [48] (v.0.11.5) was used to estimate the quality of the raw reads. Reads were trimed using Trimmomatic [49] (v.0.39) (SLIDINGWINDOW: 10:20 LEADING:20 TRAILING:20 ILLUMINACLIP:adaptor.fasta:2:20:10:1:true MINLEN:36) before downstream analysis. Bismark [50] (v. 0.22.1) was used to align reads to the human reference genome (hg38, -X 700). The human reference genome was first transformed into a bisulfite-converted version (C-to-T and G-to-A converted) and then indexed using bowtie2 [51] (v.2.4.1). Sequence reads were also transformed into fully bisulfite-converted versions before they were aligned to the genome in a directional manner. Reads that produced a unique best alignment from the two alignment processes (original top and bottom strand) were then compared to the original genomic sequence, and the methylation state of each cytosine position in the read was inferred. Duplicated reads were removed before calculating sequencing depth and coverage. bedGraph files from bismark_methylation_extractor (bismark_methylation_extractor –no_overlap -p –comprehensive –cytosine_report –CX_context) were converted to bigWig format using bedGraphToBigWig function from the ucsctools (v.3.7.8). The bisulfite conversion rate (beta value) was calculated as the percentage of thymine sequenced at cytosine reference positions in the lambda genome.

### Differentially Methylated Regions (DMRs) detection

Differentially methylated regions (DMRs) between the hypoxic and normoxic samples were identified using DSS [52] (v. 2.40.0). Only methylation sites with read counts higher than 5 in at least one sample were retained as input for DSS. Differential methylation of CpGs between each hypoxia and matched normoxic sample was first statistically tested without replicates using the following command and parameters: DMLtest (smoothing = TRUE, smoothing.span = 500). Then, a stringent set of DMRs was identified using the following command and parameters: callDMR (delta = 0.2, p.threshold = 10 − 16, minlen = 200, minCG = 5, dis.merge = 50, pct. sig = 0.5).

### DMRs annotation and enrichment analysis

The genomic annotations of differentially methylated regions were obtained using the R packages annotatr [53] (v.1.18.0), TxDb.Hsapiens.UCSC.hg38.knownGene (v.3.13.0) and org.Hs.eg.db (v.3.13.0) from Bioconductor [54, 55]. To assess whether DMRs are enriched or depleted in the annotated regions, association analysis was performed by regioneR [56] (v.1.24.0) with a permutation test (1,000 iterations). *P*-value of 0.05 was used as a cut-off for significance.

### Development of methylation hypoxia signature score

To create a robust methylation hypoxia signature, we first refine the identified DMRs using the CPCG cohort: Samples were median dichotomized according to a Ragnum score [9] calculated from RNA-seq data and only hyper-DMRs with an averaged higher methylation in the high score group were retained. The average methylation levels of the overlapping hyper-DMRs were used to develop methylation hypoxia signature score (Fig. 3).

### Survival analysis

Kaplan–Meier plots were created using BoutrosLab.plotting.general [57] (v.5.9.8) and BoutrosLab.plotting.survival (v.3.0.10), in which comparisons of survival between two groups were calculated using a log-rank test (cut-off *p*-value = 0.05). Progression-free (PFS) survival was used in the TCGA cohort, and overall survival (OS) was used in the WCDT cohort. The biochemical recurrence-free (BCR) survival was used for cases from CPCG and CPGEA cohorts.

### Code availability

All R packages used are available online as described in the method section.

### Quantification and statistical analysis

Statistical analyses were performed using R statistical environment (v3.6.1) (R Core Team, 2019) unless otherwise stated. All tests were two-sided unless otherwise specified. The different clinical features distribution across the BCR event is evaluated and significance is determined using the Wilcoxon rank sum test and Fisher's exact test. The analyses of PIMO pattern and PIMO intensity association with different clinical features were performed in R 4.0.3. The clinical and PIMO features are transformed into three data types namely categorical, binary and continuous (Supplementary Table 4). In this study, all features with binary values are ordered. The association analysis technique used varied with the types of data types compared: Spearman rank correlation for non-categorical variables, Cramer V association and Fisher’s exact test for categorical variables and correlation ratio, and Kruskal–Wallis Rank Sum Test for categorical and numerical features. PIMO intensity association with time to BCR event is determined by a univariate cox proportional hazard model. PIMO intensity is used as a continuous feature as well as binary feature. For all clinicopathologic and clinical outcomes analyses, statistical significance was defined as *p*-value < 0.05.

### Supplementary Information

Supplementary Material 1.

## Data Availability

The datasets generated in the current study are available in the European Genome-phenome Archive with accession number EGAC00001000912. Further information and requests for resources should be directed to and will be fulfilled by Lead Contact, Housheng Hansen He (hansenhe@uhnresearch.ca).
