# Supplementary material for: Oral pimonidazole unveils clinicopathologic and epigenetic features of hypoxic tumour aggressiveness in localized prostate cancer
Source: BMC Cancer. 2024 Jun 18;24:744. doi: 10.1186/s12885-024-12505-1 (PMC11186205; doi:10.1186/s12885-024-12505-1)
Supplement: Supplementary file 1 — Supplementary Material 1. [file 12885_2024_12505_MOESM1_ESM.docx]

#### **Supplemental material**


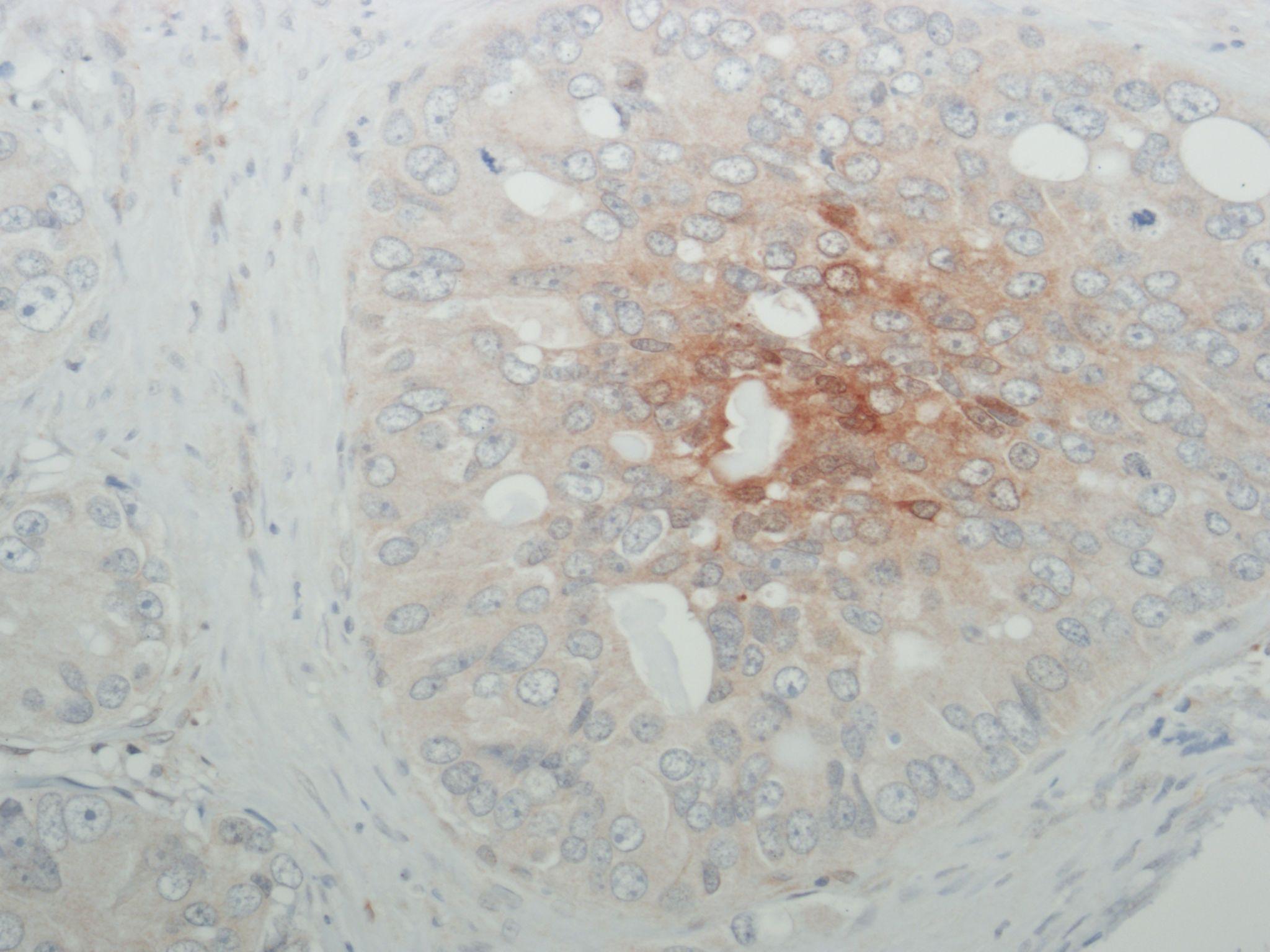


**Figure S1.**

Representative image of PCa cribriform architecture (sheet of carcinoma cells with punched-out lumina and no intervening stroma) with central staining of tumor cells defining the comedo-like PIMO staining pattern.

####
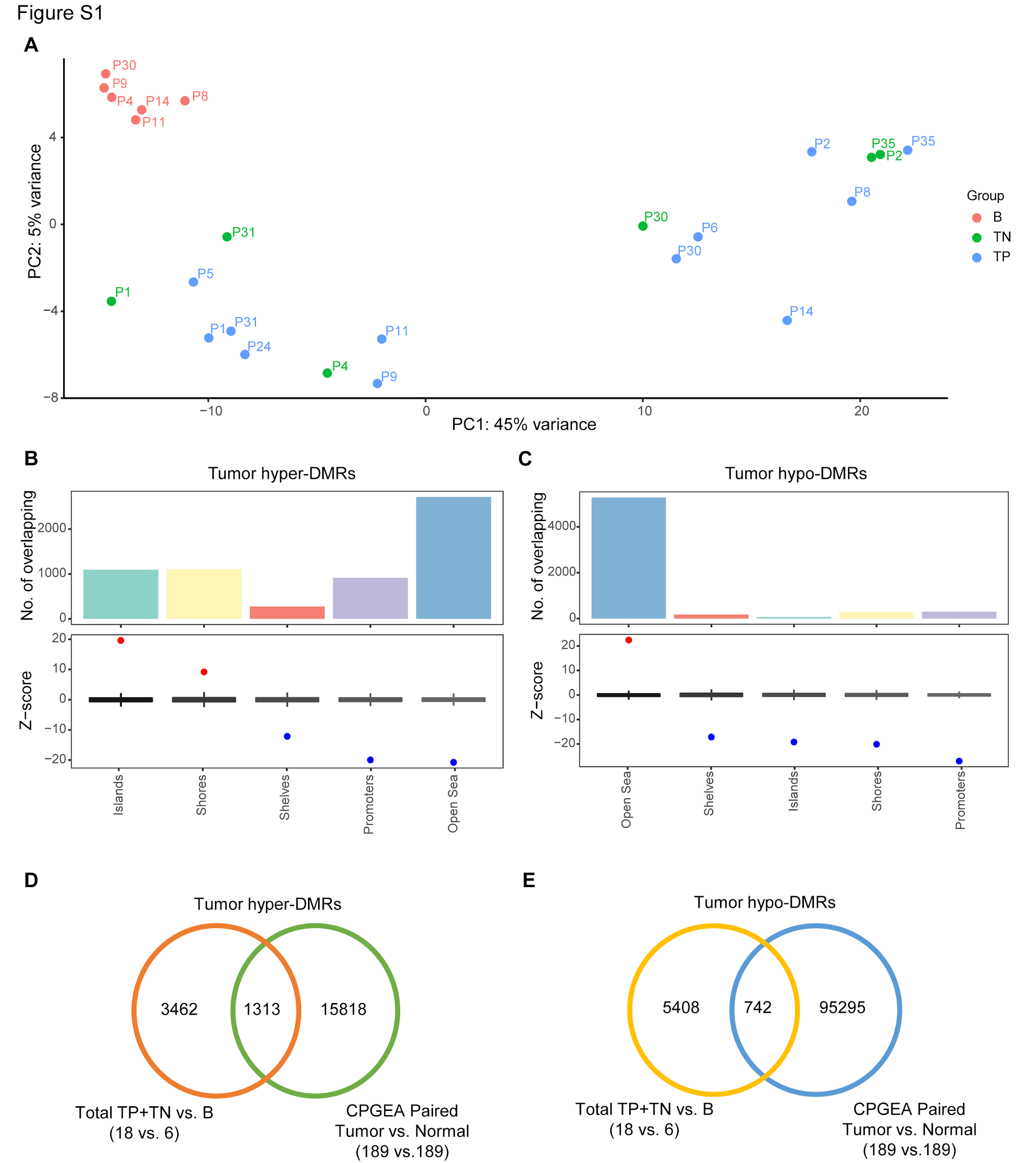


#### **Figure S2. Related to Figure 2.**

**A**) Principal component analysis of DNA methylation in all benign and tumor samples. B, Benign, TP, tumor PIMO positive; TN, tumor PIMO negative. Annotation of the tumor specific hyper- **B**) and hypo- **C**) DMRs on genomic regions. Comparison of the tumor vs. normal hyper- **D**) and hypo- **E**) DMRs identified with the current WGBS data and that from the previous CPGEA cohort. DMRs for the current study were identified by comparing all TP + TN samples to Benign.

####
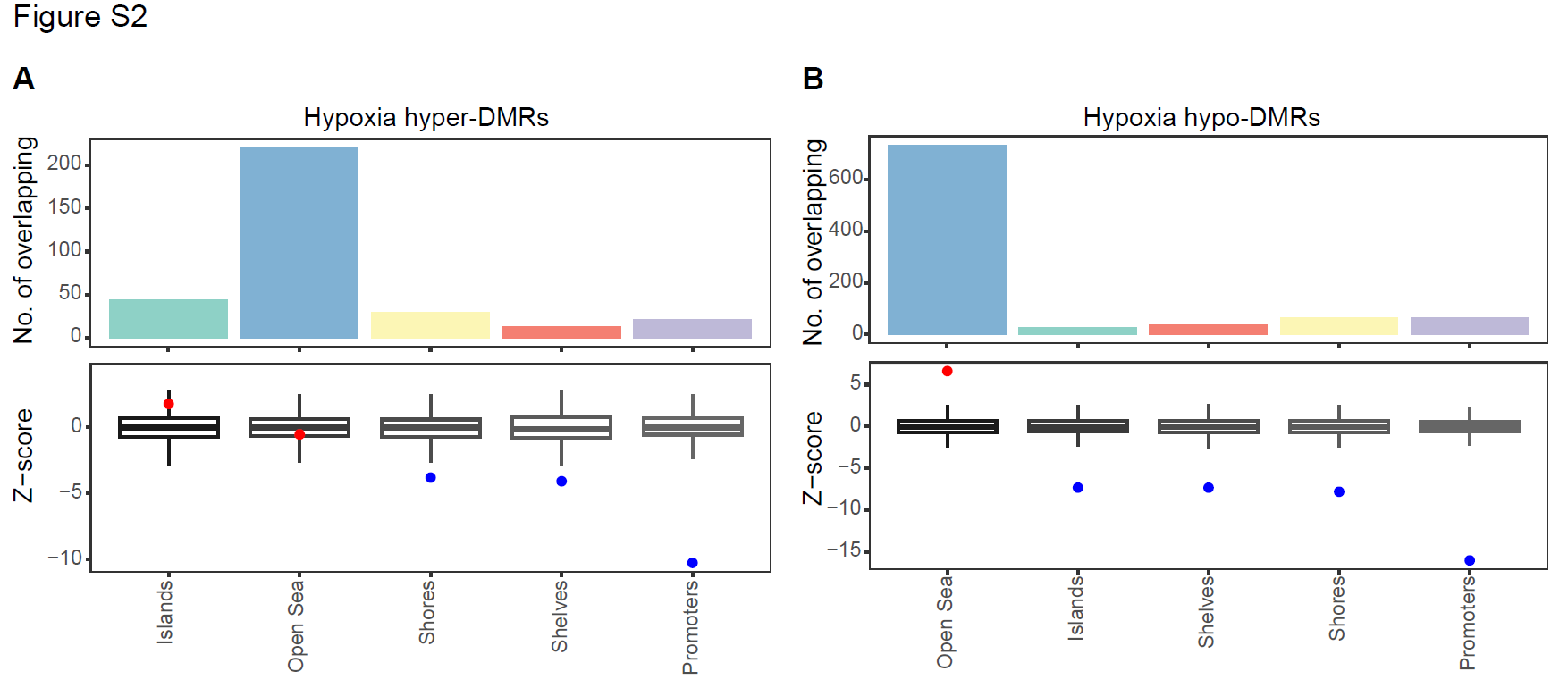


#### **Figure S3. Related to Figure 3.**

Annotation of the hypoxia specific hyper- **A**) and hypo- **B**) DMRs on genomic regions.


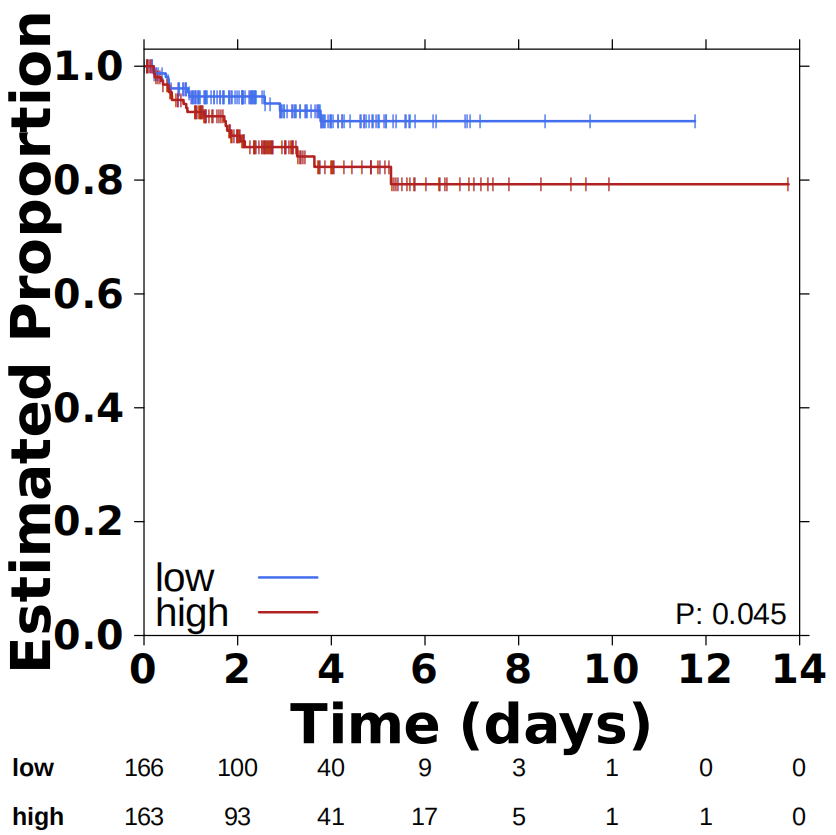


#### **Figure S4. Related to Figure 3.**

Survival rates for the high and low groups of patients, stratified using the hypoxia DMR signature score in the TCGA cohort using biochemical recurrence-free (BCR).

**Supplementary Table**

#### **Table S1. Table of patient and sampling information**

| **Patient ID** | **Gleason Score (RadP)** | **ISUP GG category** | **IDC/CA** | **PIMO IHC** | **PIMO Intensity** | **PIMO Pattern** | **LCM-WGBS** |
| --- | --- | --- | --- | --- | --- | --- | --- |
| P1 | 3+4 | LG | N | Y | 2+(HG) | Diffuse | TP, TN |
| P2 | 4+3 | HG | Y (+/+) | Y | 3+(HG) | Diffuse | TP, TN |
| P3 | 3+4 | LG | N | Y | 1+(HG) | Negative |  |
| P4 | 4+3 | HG | N | Y | negative | Negative | TN, B |
| P5 | 3+4 | LG | N | Y | 3+(LG) | Diffuse | TP |
| P6 | 5+4 | HG | Y(IDC) | Y | 3+(HG) | Comedo-like | TP |
| P7 | 3+4 | LG | N | Y | 2+(LG) | Diffuse |  |
| P8 | 4+3 | HG | Y(+/+) | Y | 3+(HG) | Comedo-like-diffuse | TP, B |
| P9 | 4+3 | HG | Y (+/+) | Y | 3+(HG) | Comedo-like-diffuse | TP, B |
| P10 | 4+3 | HG | N | Y | negative | Negative |  |
| P11 | 4+3 | HG | N | Y | 2+(HG) | Diffuse | TP, B |
| P12 | 4+4 | HG | N | Y | negative | Negative |  |
| P13 | 4+5 | HG | Y (+/+) | Y | 3+(HG) | Comedo-like-diffuse |  |
| P14 | 4+4 | HG | Y(+/+) | Y | 3+(HG) | Comedo-like-diffuse | TP, B |
| P15 | 4+3 | HG | N | Y | 2+(HG) | Focal |  |
| P16 | 4+4 | HG | Y(IDC) | Y | 1+(HG) | Negative |  |
| P17 | 3+3 | LG | N | Y | 2+(LG) | Diffuse |  |
| P18 | 3+4 | LG | N | Y | 2+(LG) | Diffuse |  |
| P19 | 4+3 | HG | N | Y | 2+(HG) | Diffuse |  |
| P20 | 3+4 | LG | N | Y | negative | Negative |  |
| P21 | 4+3 | HG | N | Y | negative | Negative |  |
| P22 | 4+4 | HG | Y(CA) | Y | 3+(HG) | Diffuse |  |
| P23 | 3+4 | LG | N | Y | negative | Negative |  |
| P24 | 4+3 | HG | Y(IDC) | Y | 3+(HG) | Diffuse | TP |
| P25 | 4+3 | HG | N | Y | negative | Negative |  |
| P26 | 3+4 | LG | N | Y | 3+(HG) | Diffuse |  |
| P27 | 4+3 | HG | Excluded | N |  | N/A |  |
| P28 | 3+3 | LG | N | Y | 3+(LG) | Focal |  |
| P29 | 4+4 | HG | Y(CA) | Y | 3+(HG) | Diffuse |  |
| P30 | 4+5 | HG | Y(IDC) | Y | 3+(HG) | Comedo-like-diffuse | TP, TN, B |
| P31 | 3+4 | LG | Y(IDC) | Y | 3+(HG) | Comedo-like-diffuse | TP, TN |
| P32 | 3+4 | LG | N | Y | 2+(LG) | Diffuse |  |
| P33 | 4+3 | HG | Y(IDC) | Y | 3+(HG) | Comedo-like-diffuse |  |
| P34 | 3+4 | LG | N | Y | 2+(LG) | Diffuse |  |
| P35 | 4+5 | HG | Y(+/+) | Y | 3+(HG) | Comedo-like-diffuse | TP, TN |
| P36 | 4+4 | HG | Y(+/+) | Y | negative | Negative |  |
| P37 | 3+4 | LG | Y(CA) | Y | negative | Negative |  |
| P38 | 4+3 | HG | Y(IDC) | Y | 2+(HG) | Diffuse |  |
| P39 | 4+3 | HG | Excluded | N |  | N/A |  |

**Table S2. Distribution of PIMO staining patterns in 37 evaluable prostatectomy specimens**

| **PIMO Pattern** | **Number of cases** |
| --- | --- |
| No staining | 11 |
| Focal | 2 |
| Diffuse – non comedo-like | 15 |
| Comedo-like | 9 |
| Total | 37 |

**Table S3. PIMO staining patterns in 17 cases with presence of IDC/CA**

| **PIMO Pattern** | **Number of cases** |
| --- | --- |
| No staining | 3 |
| Focal | 0 |
| Diffuse – non comedo-like | 5 |
| Comedo-like | 9 |
| Total | 17 |

## **Table S4. Full table of PIMO staining correlated clinical features**

| **Clinical Features** | **Value type** | **PIMO Pattern (Categorical)** | | | **PIMO Intensity (Numerical)** | | |
| --- | --- | --- | --- | --- | --- | --- | --- |
|  |  | **Association** | **p-value** | **n** | **Association** | **p-value** | **n** |
| Age at diagnosis | Numerical/Binary | 0.04 | 0.696 | 37 | -0.08 | 0.627 | 37 |
| BCR at 5 years | Numerical/Binary | 0.26 | 0.121 | 17 | 0.45 | 0.069 | 17 |
| Clinical T-category | Categorical | 0.27 | 0.539 | 37 | 0.1 | 0.179 | 37 |
| Pathologic T-category | Categorical | 0.56 | 0.005 | 37 | 0.11 | 0.047 | 37 |
| RP primary Gleason pattern | Numerical/Binary | 0.22 | 0.055 | 37 | 0.04 | 0.819 | 37 |
| RP secondary Gleason pattern | Numerical/Binary | 0.04 | 0.153 | 37 | 0.45 | 0.006 | 37 |
| RP sum Gleason score | Numerical/Binary | 0.27 | 0.019 | 37 | 0.48 | 0.003 | 37 |
| RP ISUP Grade group | Numerical/Binary | 0.12 | 0.233 | 37 | 0.13 | 0.442 | 37 |
| Percent of tumor burden | Numerical/Binary | 0.11 | 0.094 | 37 | 0.19 | 0.252 | 37 |
| Extraprostatic extension | Numerical/Binary | 0.31 | 0.011 | 37 | 0.34 | 0.038 | 37 |
| LVI | Numerical/Binary | 0.49 | 0.001 | 37 | 0.41 | 0.011 | 37 |
| Nodal status | Categorical | 0.43 | 0.062 | 36 | 0.03 | 0.26 | 36 |
| Positive margins | Numerical/Binary | 0.13 | 0.204 | 37 | 0.29 | 0.087 | 37 |
| Number of positive margins | Numerical/Binary | 0.41 | 0.071 | 14 | 0.58 | 0.031 | 14 |
| PSA at diagnosis | Numerical/Binary | 0.09 | 0.175 | 37 | 0.14 | 0.422 | 37 |
| PSA pre RadP | Numerical/Binary | 0.07 | 0.261 | 37 | 0.15 | 0.361 | 37 |
| PSA pre RT | Numerical/Binary | 0.05 | 0.968 | 13 | -0.09 | 0.778 | 13 |
| PIN | Numerical/Binary | 0.05 | 0.617 | 37 | 0.03 | 0.878 | 37 |
| IDC/CA | Categorical | 0.63 | 0.001 | 37 | 0.2 | 0.002 | 37 |

## **Table S5. Association between comedo-like PIMO pattern and adverse pathology**

| **Variable** | **Adverse Pathology** | **Odds Ratio (95% CI)** | **p-Value** | **n** |
| --- | --- | --- | --- | --- |
| Comedo (Ref = Diffuse/Other) | pTstage (III) | 13.57 (1.33,1851) | **0.024** | 26 |
| Comedo (Ref = Diffuse/Other) | LVI (1) | 15.86 (2.13-202) | **0.006** | 26 |

## **Table S6. WGBS statistics**

| **Sample** | **Total Reads (M)** | **UniqMapped (M)** | **Uniq%** | **Deduped (M)** | **Deduped%** | **Total C** | **Total mCpG (M)** | **Total mCpG (%)** |
| --- | --- | --- | --- | --- | --- | --- | --- | --- |
| TP1 | 357.13 | 280.04 | 78.42% | 240.58 | 85.91% | 6963.46 | 260.86 | 77.00% |
| TN1 | 396.49 | 311.36 | 78.53% | 254.47 | 81.73% | 7076.45 | 266.61 | 78.40% |
| TP2 | 686.62 | 536.94 | 78.20% | 404.34 | 76.00% | 11730.43 | 407.1 | 68.60% |
| TN2 | 513.79 | 405.64 | 78.95% | 317.65 | 78.31% | 9131 | 296.06 | 66.40% |
| TN4 | 349.83 | 276.47 | 79.03% | 221.21 | 80.01% | 6626.05 | 233.54 | 75.70% |
| B4 | 389.9 | 309.65 | 79.42% | 237.89 | 76.83% | 7170.93 | 261.31 | 77.30% |
| TP5 | 361.37 | 276.54 | 76.53% | 213.89 | 77.34% | 6226.6 | 236.71 | 76.80% |
| TP6 | 343.65 | 268.13 | 78.02% | 211.21 | 78.77% | 6179.95 | 214.62 | 66.00% |
| TP8 | 349.25 | 274.42 | 78.57% | 219.14 | 79.86% | 6860.7 | 229.89 | 65.50% |
| B8 | 388.35 | 303.35 | 78.11% | 235.14 | 77.51% | 7202.23 | 276.35 | 75.50% |
| TP9 | 352.72 | 275.44 | 78.09% | 208.11 | 75.55% | 5969.29 | 203.7 | 74.00% |
| B9 | 303.95 | 236.05 | 77.66% | 188.6 | 79.90% | 5214.18 | 186.73 | 76.90% |
| TP11 | 304.19 | 242.63 | 79.76% | 197.84 | 81.54% | 6128.09 | 224.48 | 74.50% |
| B11 | 336.37 | 266.44 | 79.21% | 221.26 | 83.04% | 6527.58 | 240.28 | 76.90% |
| TP14 | 340.1 | 269.34 | 79.19% | 213.29 | 79.19% | 6406.2 | 197.39 | 60.60% |
| B14 | 357.16 | 284.79 | 79.74% | 240.7 | 84.52% | 7355.98 | 279.22 | 74.90% |
| TP24 | 323.6 | 257.69 | 79.63% | 213.94 | 83.02% | 6254.39 | 206.24 | 73.90% |
| TP30 | 397.1 | 317.52 | 79.96% | 203.56 | 64.11% | 5760.71 | 190.97 | 70.60% |
| TN30 | 447.09 | 353.56 | 79.08% | 214.72 | 60.73% | 5941.37 | 186.64 | 70.60% |
| B30 | 309.16 | 247.57 | 80.08% | 201.19 | 81.27% | 5875.71 | 197.62 | 76.30% |
| TP31 | 322.92 | 264.24 | 81.83% | 217.72 | 82.39% | 7241.22 | 251.43 | 74.20% |
| TN31 | 342.69 | 276.09 | 80.57% | 224.68 | 81.38% | 7045.46 | 251.64 | 75.00% |
| TP35 | 345.78 | 276.79 | 80.05% | 226.37 | 81.78% | 7052 | 222.07 | 65.20% |
| TN35 | 347.12 | 278.25 | 80.16% | 225.7 | 81.11% | 7244.03 | 248.18 | 67.50% |

##

##

##

##

##

## **Table S7. Association between other hypoxia scores and disease outcome**

| **Various scores** | **P value** | | | |
| --- | --- | --- | --- | --- |
|  | **TCGA** | **CPCG** | **CPGEA** | **WCDT** |
| Ragnum RNA Scores | 0.65 | 0.061 | 0.0036 | 0.13 |
| hyperDMR Scores | 0.054 | 0.094 | 0.014 | 0.11 |
| hypoDMR Scores | 0.082 | 0.31 | 0.042 | 0.95 |

## Ragnum RNA Scores: 32 hypoxia genes; hyperDMR Scores: Total sites of hyper-DMRs; hypoDMR Scores: Total sites of hypo-DMRs.

##

## **Table S8. The survival analysis of hypoxia methylation signature in other tumor types (TCGA datasets)**

| **Tumor Types** | **P value** |
| --- | --- |
| Lower Grade Glioma | 3.88*10-5 |
| Kidney Renal Clear Cell Carcinoma | 0.0044 |
| Glioblastoma Multiforme | 0.054 |
| Muscle-Invasive Bladder Cancer | 0.8 |
| Breast Invasive Carcinoma | 0.3 |
| Liver hepatocellular carcinoma | 0.9 |
| Lung Squamous Cell Carcinoma | 0.33 |
| Lung Adenocarcinoma | 0.24 |

## 
